# Topology and Nuclear Size Determine Cell Packing on Growing Lung Spheroids

**DOI:** 10.1103/physrevx.15.011067

**Published:** 2025-03-21

**Authors:** Wenhui Tang, Jessie Huang, Adrian F. Pegoraro, James H. Zhang, Yiwen Tang, Darrell N. Kotton, Dapeng Bi, Ming Guo

**Affiliations:** 1 Department of Mechanical Engineering, Massachusetts Institute of Technology, Cambridge, Massachusetts, USA; 2 Center for Regenerative Medicine of Boston University and Boston Medical Center, Boston, Massachusetts, USA; 3 Metrology Research Centre, National Research Council Canada, Ottawa, Ontario, Canada; 4 Department of Physics, Northeastern University, Boston, Massachusetts, USA; 5 Center for Theoretical Biological Physics, Northeastern University, Boston, Massachusetts, USA

**Keywords:** Biological Physics, Interdisciplinary Physics

## Abstract

Within multicellular living systems, cells coordinate their positions with spatiotemporal accuracy to form various tissue structures and control development. These arrangements can be regulated by tissue geometry, biochemical cues, as well as mechanical perturbations. However, how cells pack during dynamic three-dimensional multicellular architectures formation remains unclear. Here, examining a growing spherical multicellular system, human lung alveolospheres, we observe an emergence of hexagonal packing order and a structural transition of cells that comprise the spherical epithelium. Surprisingly, the cell packing behavior on the spherical surface of lung alveolospheres resembles hard-disks packing on spheres, where the less deformable cell nuclei act as effective “hard disks” and prevent cells from getting too close. Nucleus-to-cell size ratio increases during lung spheroids growth; as a result, we find more hexagon-concentrated cellular packing with increasing bond orientational order. Furthermore, by osmotically changing the compactness of cells on alveolospheres, we observe a more ordered packing when nucleus-to-cell size ratio increases, and vice versa. These more ordered cell packing characteristics are consistent with reduced cell dynamics, together suggesting that better cellular packing stabilizes local cell neighborhoods and may regulate more complex biological functions such as cellular maturation and tissue morphogenesis.

## I. INTRODUCTION

The surfaces of three-dimensional (3D) multicellular systems, such as blood vessels, intestines, and pulmonary alveoli, are densely packed with cells. How cells coordinate and optimize their relative positions is informative in various physiological and pathological processes [1–7]. For example, cell shapes change drastically during the extension of *Drosophila* germband epithelium [4] and pupal wing formation [8]; in breast cancer clusters, cells on the boundary become larger and more elongated when forming invasive protrusions [5]. Moreover, cell nearest neighbor order changes in conjunction with the phase of tissues during jamming or glass transitions in a variety of simulation and experimental studies [2,3,9]. Despite these findings on how cell shape varies during different processes, it still remains relatively unclear what physical mechanisms regulate cell packing to tile tissue surfaces, and how cell packing may influence tissue morphogenesis and function. In contrast to monodispersed colloidal systems, packing in living tissues is highly disordered due to their compressibility and variability in size. In addition, tissue and organ surfaces are naturally curved in 3D with nonzero Gaussian curvatures [10–14], which adds another level of complexity to the packing problem. Although recent studies have shown that cell and tissue morphology can be affected by a variety of factors, such as cell density [6,15–17], curvature [16], nuclear shape [16,17], and mechanical forces [6], it still remains unclear how soft cells tile a curved tissue surface and how cell packing is regulated, particularly during growth and maturation of 3D living systems. Here, using human induced pluripotent stem cell (iPSC) derived lung alveolospheres as a model system, we observe an emergence of packing order, specifically a structural gas-to-liquid transition, during the growth of lung alveolospheres, determined by the increasing nucleus-to-cell size ratio and topology. As cell packing order increases, these spherical epithelia become less dynamic and more stable. Our finding reveals the critical role of cell nuclear size in regulating cell packing order during tissue development, suggesting the importance of structural phase changes in establishing tissue stability.

**FIG. 1.**
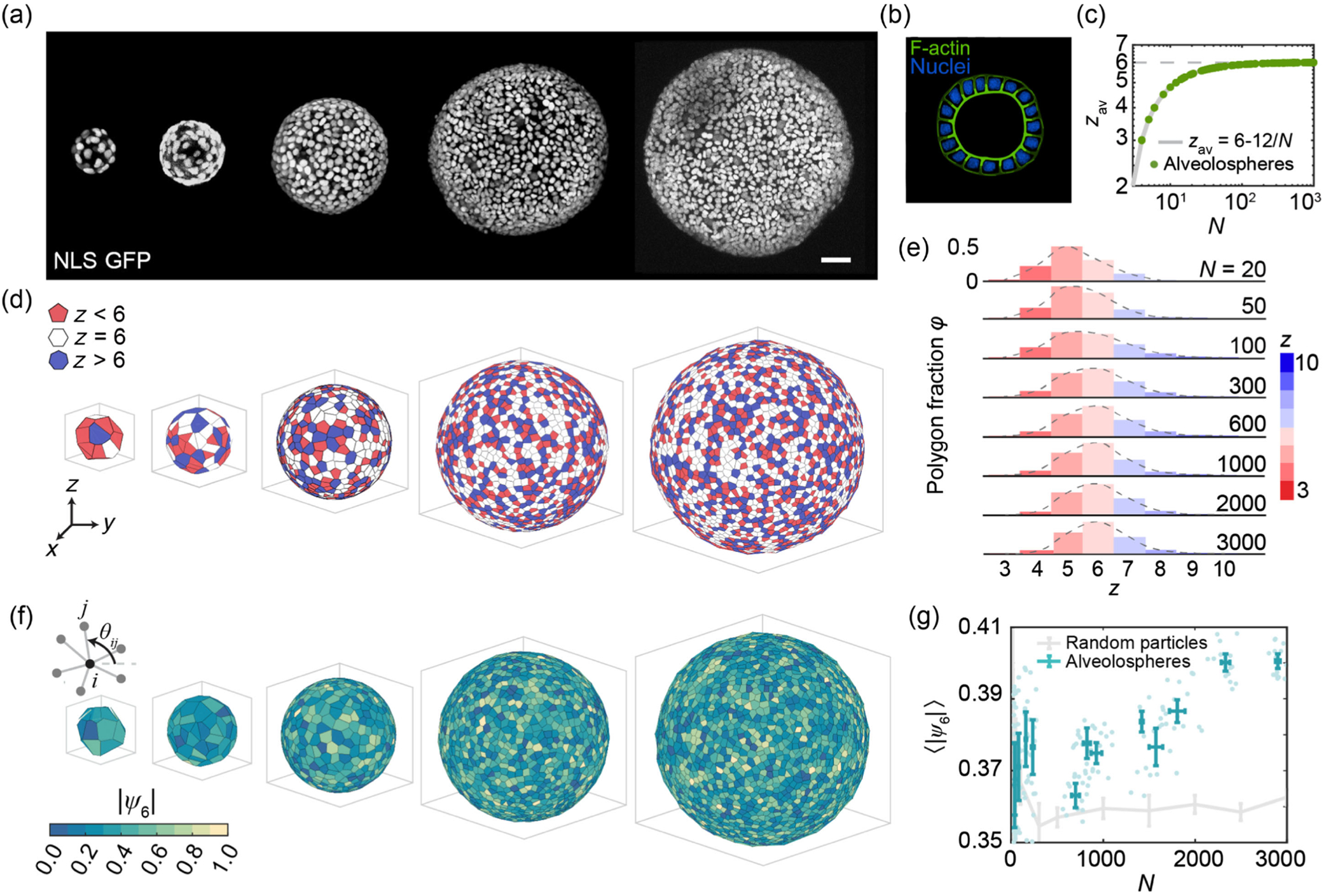
Emergence of topological packing order on growing human lung alveolospheres. (a) 3D projections of GFP NLS nuclei on representative growing alveolospheres. Scale bar: 50μm. (b) Schematics of cross-sectional image for an alveolosphere. (c) Average vertex number $z_{\text{av}}$ of all cells on each alveolosphere as a function of total cell number N (green dots) follows the theoretical curve $z_{\text{av}}=6-12/N$ (gray curve), as shown in a log-log plot. (d) Voronoi tessellation shows cell nearest neighbor order with three categories (z<6,z=6,z>6) for alveolospheres with increasing total cell number N. (e) The distribution of different cell shapes showing that hexagonal cells become the predominant population as alveolospheres grow bigger. (f) Bond orientational order $\left|\psi_{6}\right|$ over each alveolosphere for representative alveolospheres. (g) Average bond orientational order $\left\langle |\psi_{6}|\right\rangle$ of lung alveolospheres (cyan) continues to increase as N increases, despite $z_{\text{av}}$ remaining almost constant. Average bond orientational order of random particle simulation (gray) shows that $\left\langle |\psi_{6}|\right\rangle$ almost remains constant with increasing N. Mean ± s.d. are shown in both curves.

To study the packing of cells on curved biological surfaces, we use a human lung alveolosphere system cultured in 3D in Matrigel; this system contains only a monolayer of human iPSC-derived alveolar epithelial type II cells (iAT2s) and exhibits a hollow spherical geometry [18], as shown in the cross-sectional image Fig. 1(b). These iAT2s express a global transcriptome and ultrastructure that resemble primary adult alveolar epithelial type II cells (AT2s), thus serving as an *in vitro* model of AT2-related human lung development. As these alveolospheres grow in size, the total cell number within an alveolosphere increases, thus allowing us to investigate the packing order of the constituted iAT2s on these evolving spherical biological surfaces. To visualize cells in alveolospheres, we transduce them with a lentiviral vector to stably express green fluorescent protein fused to a nuclear localization signal (GFP NLS) allowing nuclear localization of green fluorescence. With this system we monitor individual cell positions and behavior in each hollow sphere by imaging entire alveolospheres using confocal microscopy [19]. We then track the positions of individual cell nuclei in 3D for alveolospheres of different sizes, and obtain the packing order of cells on these spherical surfaces.

## II. TOPOLOGICAL CONSTRAINT AND EMERGENCE OF PACKING ORDER ON GROWING LUNG SPHEROIDS

Using the nuclear positions in alveolospheres (iPSC clone BU3 NGST), we obtain cell nearest neighbor orders by performing Voronoi tessellation on the spherical surface [Fig. 1(d)] [20,21]. Nuclear centers are tracked in 3D and then projected onto the surface of a unit sphere to get Voronoi polygons. Each cell nucleus generates one Voronoi polygon, whose vertex number $z_{i}$ corresponds to the number of nearest neighbors for each cell. Each polygon represents the nearest neighbor order for the corresponding cell; the composition of polygons shows the global packing order on each alveolosphere. Hexagons ($z_{i}=6$), pentagons ($z_{i}=5$), and heptagons ($z_{i}=7$) are among the most common nearest neighbor orders. Compared to the packing of monodispersed colloidal particles on spheres [10,12,22–24], we find a significantly larger amount of nonhexagons with living biological cells on lung spheroids. Yet cell packing still obeys simple topological constraints. This is because in Voronoi tessellation, the facets typically meet at vertices where three cells converge, and each edge is shared by two facets, and thus the Euler’s polyhedron formula results in the average cell nearest neighbor order $z_{\text{av}}$ to be $z_{\text{av}}=6-12/N$ on spherical biological surfaces (Appendix B) [25–27], as shown in Fig. 1(c). The cell packing on alveolospheres becomes more ordered as they grow bigger: Hexagons gradually replace pentagons as the predominant cell nearest neighbor order with increasing alveolosphere size N [Fig. 1(e)]. Interestingly, although the hexagon fraction plateaus at large N (Fig. 5), we observe a slight increase in bond orientational order $\psi_{6}$ as N increases [Appendix G; see Fig. 1(g)]; this increase is noticeable compared to the $\psi_{6}$ of random particles on the spheres at various N, which suggests that the rise in $\psi_{6}$ exceeds the effect of local angle deficit caused by the finite integrated Gaussian curvature in each facet. This increase in $\psi_{6}$ reveals a higher level of structural regularity, with the average cell packing order being closer to regular hexagons (with 120 deg angle at each vertex) [12,14]. Interestingly, if we compare the nearest neighbor order of cell packing in alveolospheres to a random particle-on-sphere simulation, both plateau at large N, but alveolosphere packing has a higher ratio of hexagons than random packing (Fig. 5). This difference in nearest neighbor order suggests that cell packing on alveolospheres is not random, and the arrangement of cells within spherical epithelia is influenced by factors beyond just spherical topological constraint.

## III. NUCLEI SET THE MINIMUM CELL-CELL DISTANCE AND DETERMINE PACKING ORDER

**FIG. 2.**
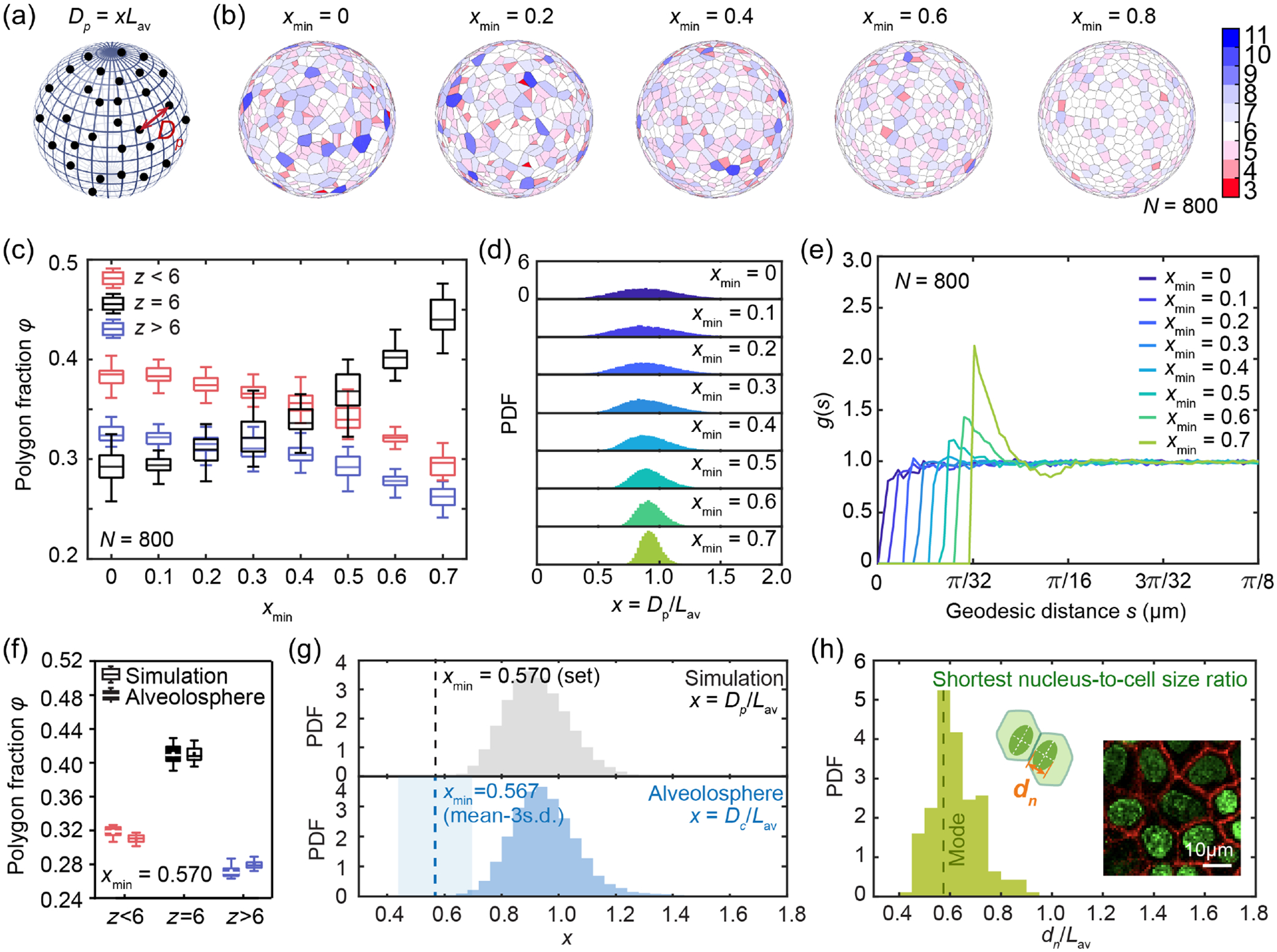
Nucleus-to-cell size ratio regulates nearest neighbor order on alveolospheres. (a) Schematics of particles-on-sphere simulation, with the distance between particles defined as $D_{p}=xL_{\text{av}}$, where $D_{p}$ is the average particle size and x is a ratio. (b) Increasing $x_{\text{min}}$ in simulation results in a higher fraction of hexagons and more ordered packing on a unit sphere with N=800 in simulation. (c) Quantification of nearest neighbor order fractions in simulation shows that as $x_{\text{min}}$ increases, the proportion of z=6 increases while the proportion of z<6 and z>6 decreases. Each box plot is from 30 tests of simulation. (d) Varying $x_{\text{min}}$ in simulation changes the distribution of nondimensionalized particle-particle distance $D_{p}/L_{\text{av}}$ on spheres. (e) Radial distribution function g(s) as a function of geodesic length s for different $x_{\text{min}}$ when N=800 on a unit sphere. Each curve is the average of 30 datasets generated by simulation. (f) Nearest neighbor order fractions for alveolospheres compared to particle simulations, with $x_{\text{min}}=0.570$ chosen for the best match. (g) Probability density function (PDF) of x in alveolospheres agrees well with simulation when $x_{\text{min}}=0.570$. Blue shaded region marks the region of (mean-3s.d.) ± s.d., which is 0.567 ± 0.129. (h) Probability density function of the shortest nucleus-cell size ratio for alveolosphere. The mode of the distribution is marked as the dashed line as 0.575.

To investigate what determines the distribution of packing order for large systems, we perform a simulation to generate N particles on a unit sphere and limit the geodesic distance between neighbor particles to be no less than a minimum distance ${\left(D_{p}\right)}_{\text{min}}$ [Fig. 2(a); see Appendix E]. To allow comparison between different total particle number N, as well as between results from simulation and experiments, this minimum distance ${\left(D_{p}\right)}_{\text{min}}$ is nondimensionalized by the average occupied length scale $L_{\text{av}}$ for each particle and is defined as

(1) $$x_{\text{min}}={\left(D_{p}\right)}_{\text{min}}/L_{\text{av}},$$

where $L_{\text{av}}=\sqrt{\left(4\pi R^{2}\right)/N\times (2/\sqrt{3})}$ is calculated based on the average area each particle occupies. If we treat this limitation of particle distance as an effective diameter of a hard disk centered at each particle, $x_{\text{min}}$ then sets the area fraction of an effective hard-disk packing, which is given by $\phi =(\pi /2\sqrt{3})x_{\text{min}}^{2}$ (Appendix D). Therefore, by changing $x_{\text{min}}$ we can vary the effective hard-disk packing fraction ϕ. We then use Voronoi tessellation to get the nearest neighbor order for all the particles on sphere and categorize Voronoi polygons into z<6,z=6,z>6. In simulation, when $x_{\text{min}}$ increases from 0 to 0.7 for the same system size (e.g., N=800), we observe that the particle packing becomes more ordered [Fig. 2(b)]; Voronoi-tessellated hexagons (z=6) gradually become the predominant population among all polygons and both the fraction of z>6 and z<6 decrease [Fig. 2(c)].

To investigate how $x_{\text{min}}$ regulates the packing order on a sphere, we examine the particle-particle distance distribution under different $x_{\text{min}}$. The distribution of particle-particle distance becomes narrower as $x_{\text{min}}$ increases [Fig. 2(d)], and this more homogeneous particle-particle distance yields a more ordered particle packing, which is consistent with recent study [28]. When comparing simulation results to experiments, we find that when $x_{\text{min}}\approx 0.57$, the nearest neighbor order compositions (z<6,z=6,z>6) are similar to those observed in alveolospheres with the same system size [Fig. 2(f)]. This suggests that a limit of cell-cell distance also regulates the nearest neighbor order in alveolospheres. To further explore this, we plot the distribution of the nondimensional cell-cell distance $x=D_{c}/L_{\text{av}}$ where $D_{c}$ is neighbor cell-cell distance [Fig. 2(g)]. Interestingly, we find that the histogram of x in alveolospheres resembles that from simulation with a preset $x_{\text{min}}=0.57$ [Fig. 2(g)]. These results suggest the existence of a minimum nondimensional cell-cell distance $x_{\text{min}}=0.57$ which regulates the packing order on this alveolosphere. One estimate of this experimental $x_{\text{min}}$ is to take the mean-3s.d. from the distribution x on the alveolosphere which we find is 0.567 ± 0.129. To test if an experimental $x_{\text{min}}$ is a universal feature of alveolospheres, we perform the same analysis on alveolospheres derived from a different donor (iPSC clone SPC2-ST-B2 [28]; see Appendix A) that naturally have smaller nuclear size; we observe that the cell packing is determined by minimum cell-cell distance as well (Fig. 7). These comparisons together show that the nearest neighbor order is regulated by the minimum cell-cell distance in lung alveolospheres.

Within epithelia, cells cannot get infinitely close due to physical limitation. Given that the nucleus is the largest organelle in eukaryotic cells and is considerably stiffer than cytoplasm [27], we wonder if cell nuclear size plays a major role in regulating the cell-cell distance and coordinating the nearest neighbor order. In alveolosphere systems, cell nuclear shape is not circular but elliptical (Fig. 6); therefore, the closest cell-cell distance is more likely to be limited by the shortest nuclear dimension $d_{n}$ of the ellipse projected on the epithelium layer [Fig. 2(h), inset]. When we measure $d_{n}$ for individual cells on alveolospheres, we can again define a nondimensionalized representation by dividing by the average cell size $L_{\text{av}}$. This allows a comparison of this dimensionless shortest nuclear dimension $d_{n}/L_{\text{av}}$ with the dimensionless cell-cell distance x, in particular its minimum value $x_{\text{min}}$ [Figs. 2(g) and 2(h)]. We find that $d_{n}/L_{\text{av}}$ has a distribution aligning well with $x_{\text{min}}$ [Fig. 2(h)], indicating that the nuclear size sets the minimum cell-cell distance. Furthermore, results of alveolospheres from a different donor (iPSC clone SPC2-ST-B2 [28]) that naturally have a smaller nuclear size confirm this finding as well (Fig. 7); a smaller nucleus-to-cell size ratio leads to a decreased cell packing order. Consistently, a recent study also shows higher nucleus area ratio results in a better packing in *in vivo* 3D *Drosophila* retina and brain [17].

**FIG. 3.**
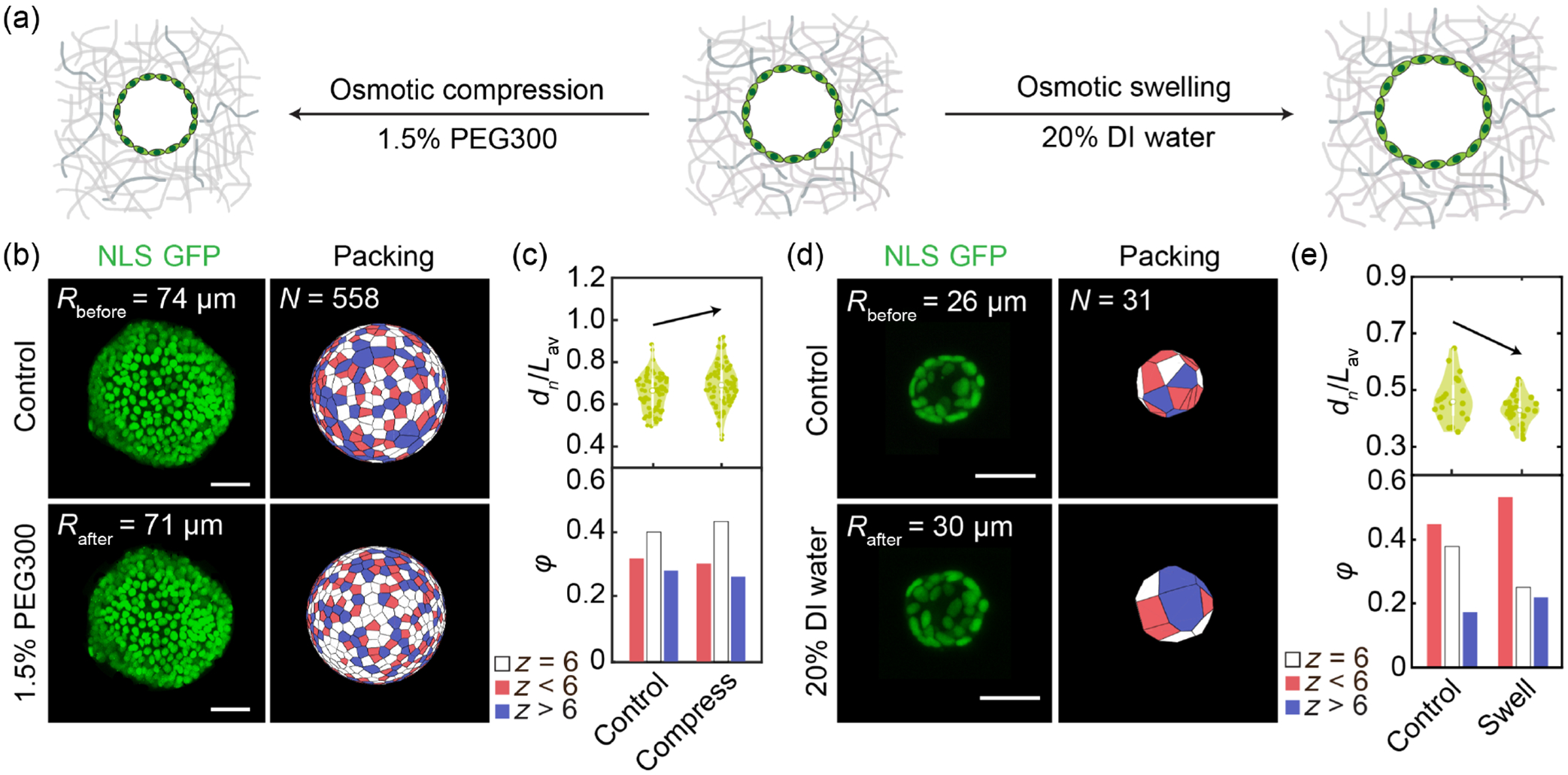
Perturbing the nucleus-to-cell size ratio changes cell packing in lung alveolospheres. (a) Schematics of osmotic compression (add 1.5% PEG300) and swelling [add 20% deionized (DI) water] to perturb alveolospheres. (b) Osmotic compression squeezes the alveolospheres, causing increased hexagon concentration in alveolospheres. Scale bars: 50μm. (c) Fractions of nearest neighbor order (bottom) and smallest nuclei ratio $d_{n}/L_{\text{av}}$ (top) evolution before and after osmotic compression. Smallest nuclei ratio $d_{n}/L_{\text{av}}$ is shown in a violin plot, where the background color is the distribution. (d) Osmotic swelling decreases hexagon concentration in alveolospheres. Scale bars: 50μm. (e) Fractions of nearest neighbor order (bottom) and smallest nuclei ratio $d_{n}/L_{\text{av}}$ evolution before and after osmotic swelling. Smallest nuclei ratio $d_{n}/L_{\text{av}}$ is shown in a violin plot, where the background color is the distribution.

To further investigate the idea that cell nuclear size sets the minimum cell-cell distance, and thus coordinates cell packing order on spherical surfaces, we apply hyperosmotic and hypo-osmotic perturbations to change relative cell nuclear size. Osmotic perturbations are performed using established methods as described in previous literature [5,29–32]. We supplement culture medium with 1.5 Vol./Vol. % polyethylene glycol 300 (PEG300) and 20 Vol./Vol. % deionized water to compress and swell the alveolospheres, respectively [Fig. 3(a); see Appendix F]. We observe an increase in relative nuclear size $d_{n}/L_{\text{av}}$ under hyperosmotic pressure and a decrease in $d_{n}/L_{\text{av}}$ under hypo-osmotic pressure [Figs. 3(e) and 8]; consistently, we find more ordered packing under osmotic compression [Figs. 3(b) and 3(c)] and less ordered packing under osmotic swelling [Figs. 3(d) and 3(e)]. Together, these results confirm that the relative cell nuclear size regulates the packing order on multicellular spheres, through limiting the minimum cell-cell distance.

## IV. NUCLEUS-TO-CELL SIZE RATIO INCREASES DURING LUNG ALVEOLOSPHERES GROWTH

**FIG. 4.**
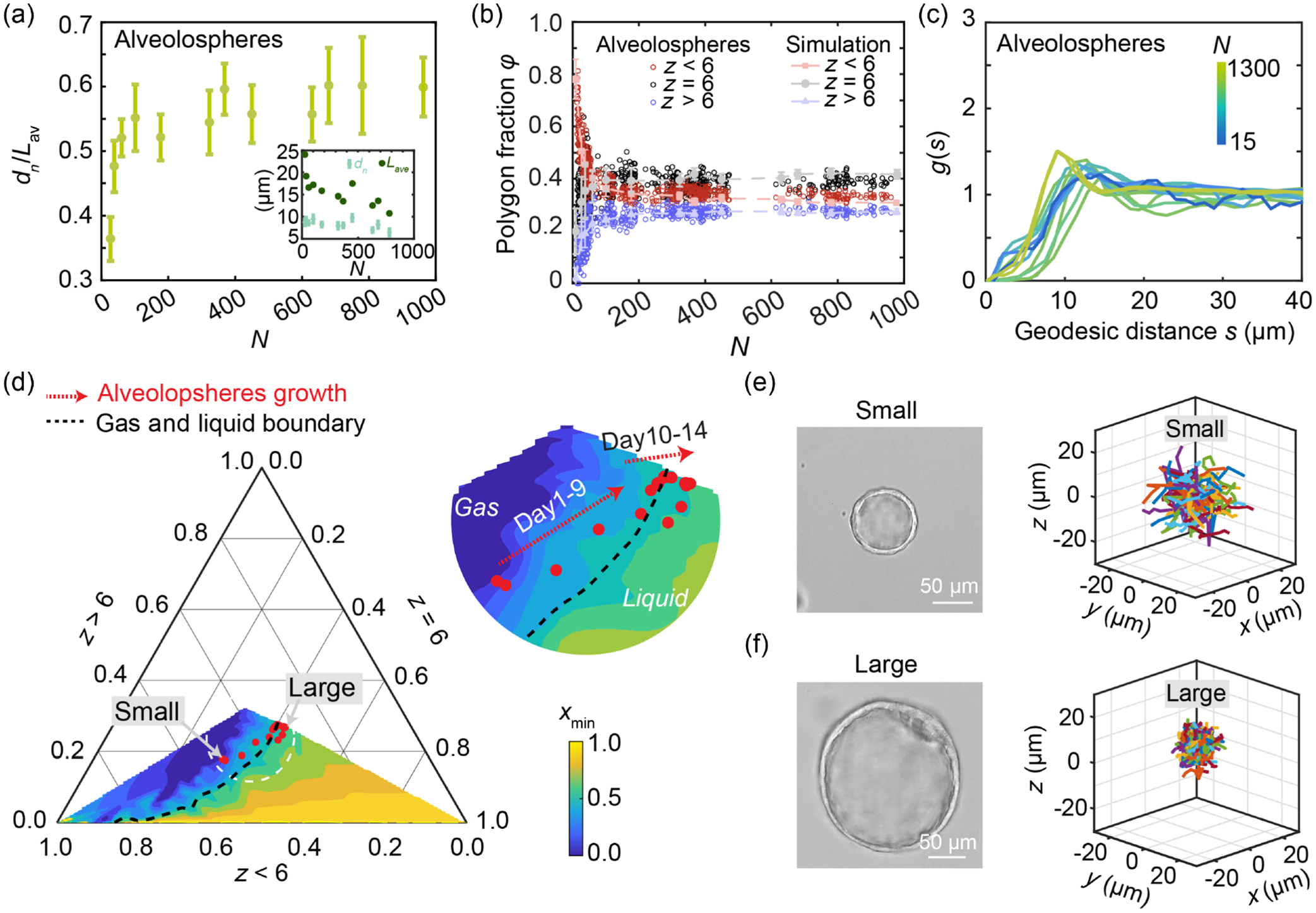
Increasing nuclear size ratio relates to a structural gas-to-liquid transition and less dynamic cell migration when alveolospheres grow bigger. (a) Experimentally measured evolution of smallest nucleus-to-cell size ratio $d_{n}/L_{\text{av}}$ as a function of the total cell number N on alveolospheres. Inset: the separate trends of the smallest nuclei dimension $d_{n}$ and average cell size $L_{\text{av}}$. (b) Simulation with the experimentally measured N and $x_{\text{min}}=d_{n}/L_{\text{av}}$ reproduces the nearest neighbor order fractions in a series of growing alveolospheres. Circles, alveolospheres data; dashed lines, simulation. (c) Radial distribution function g(s) as a function of geodesic distance s for alveolospheres with different sizes. (d) Ternary phase diagram of z<6,z=6,z>6 visualized as a function of $x_{\text{min}}$. Background color represents $x_{\text{min}}$ from particle-on-sphere simulation. Red points show the alveolospheres data. Dashed black line marks the gas-liquid phase boundary defined from the coordination number at different N (Appendix I; see Fig. 11). (e) Cell trajectories for 50 randomly selected cells within a small alveolosphere (R=30μm) during 10 continuous frames with time interval 15 min. (f) Cell trajectories for 50 randomly selected cells within the largest alveolosphere (R=160μm) during 10 continuous frames. Time interval of each frame is 15 min.

**FIG. 5.**
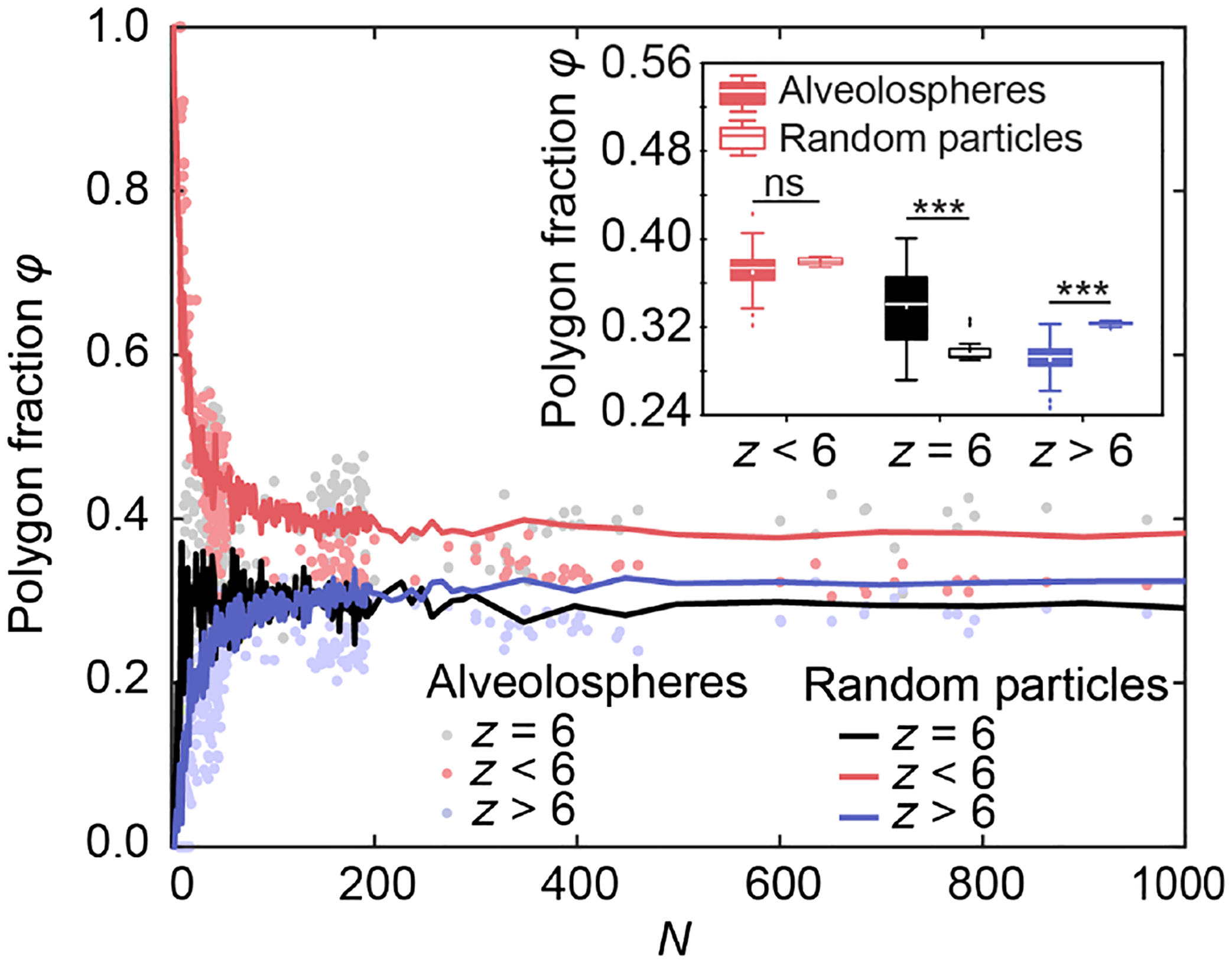
Comparison of the evolution of nearest neighbor order fractions in growing lung alveolospheres and particles randomly packed on sphere from simulation. Inset: box plot showing the three categories of nearest neighbor order fractions (z>6,z=6, and z<6) at the plateau region.

**FIG. 6.**
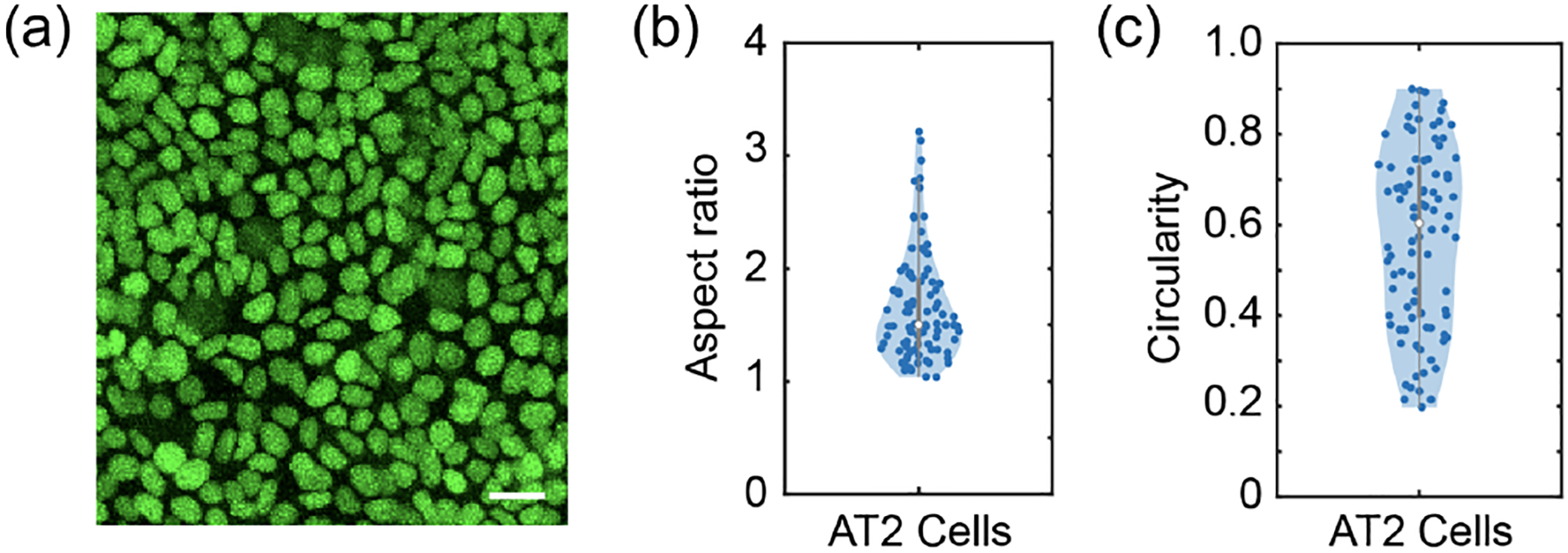
Cell nuclei in alveolospheres are not spherical but elliptical. (a) Representative cell nuclei GFP NLS showing the nucleus shape. Scale bar: 20μm. (b) Violin plot of aspect ratio for cell nuclei. (c) Violin plot of circularity for cell nuclei.

**FIG. 7.**
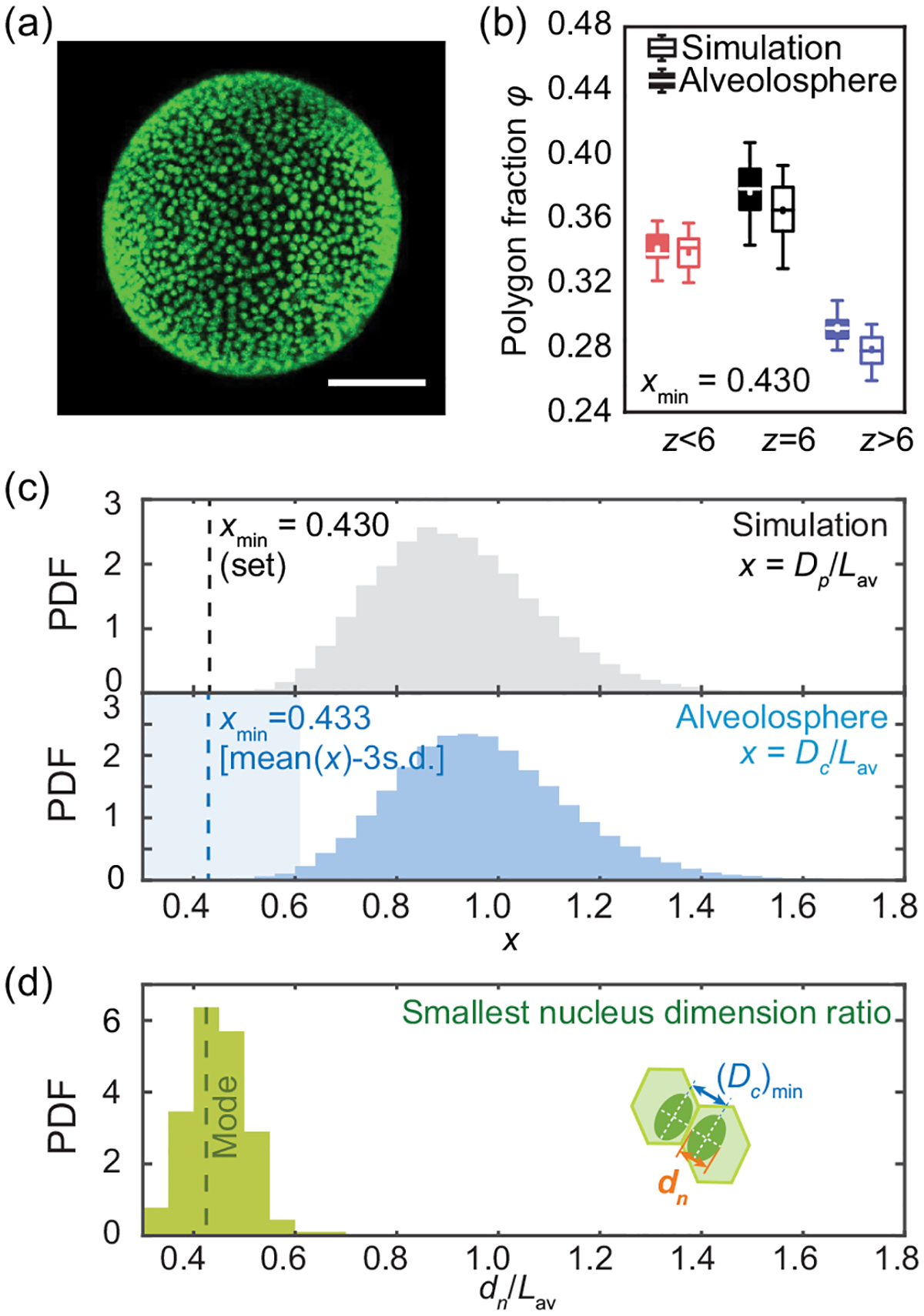
Alveolosphere (clone SPC2-B2) that naturally has a smaller nuclear-cell size ratio shows nuclear-cell size ratio aligns with the smallest cell-cell distance ratio and regulates packing fraction. (a) NLS GFP of a representative alveolosphere which has N≈1200. Scale bar: 100μm. (b) Nearest neighbor order fractions for alveolospheres compared to particle simulations for $x_{\text{min}}=0.43$ selected to best match. (c) Probability density functions (PDFs) of x in alveolosphere agrees well with particle-particle distance when $x_{\text{min}}=0.43$. Blue shade marks the region of (mean −3 s.d.) ± s.d. = 0.433 ± 0.183. (d) PDFs of the shortest nuclei ratio $d_{n}/L_{\text{av}}$ for alveolosphere. The mode of the distribution is labeled as dashed line in the middle of the bar at $d_{n}/L_{\text{av}}=0.425$.

**FIG. 8.**
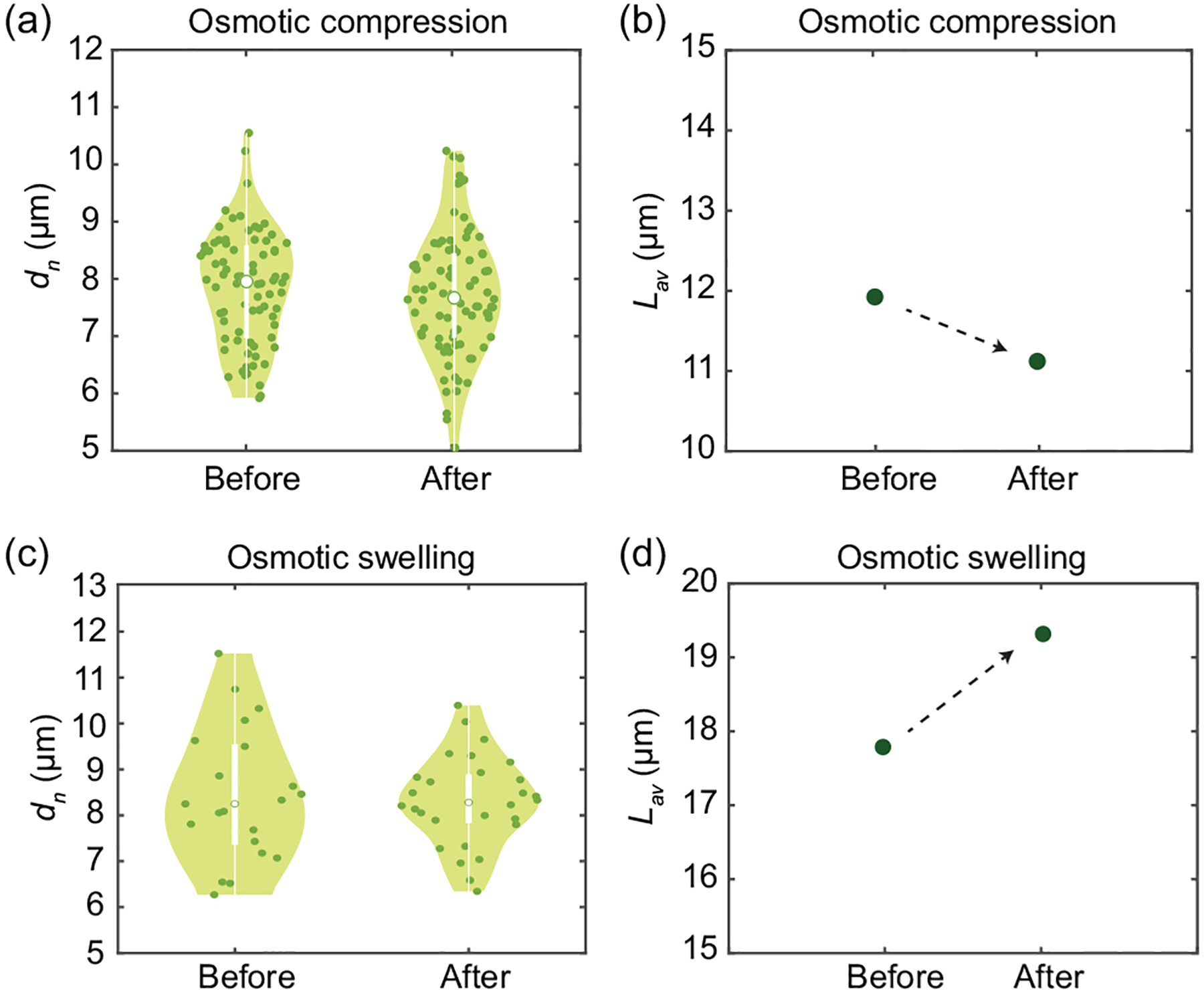
The change of $d_{n}$ and $L_{\text{av}}$ before and after osmotic compression and osmotic swelling. (a) The distribution of $d_{n}$ before and after osmotic compression. (b) $L_{\text{av}}$ (calculated based on alveolospheres radius and total cell number) decreases after osmotic compression. (c) The distribution of $d_{n}$ before and after osmotic swelling. (d) $L_{\text{av}}$ (calculated based on alveolospheres radius and total cell number) increases after osmotic swelling.

To understand how cell nuclear size dynamically regulates cellular organization on 3D curved surfaces during lung alveolosphere development, we measure the dimensionless shortest nuclear dimension $d_{n}/L_{\text{av}}$ for individual cells on alveolospheres during their growth. Remarkably, we find that $d_{n}/L_{\text{av}}$ increases during growth and seems to reach a plateau at large N [Fig. 4(a)]. While both the average cell size and nuclear size decrease with N, the former decreases faster, resulting in an increasing ratio $d_{n}/L_{\text{av}}$ as alveolospheres grow bigger [Fig. 4(a), inset]. To understand the evolution of nearest neighbor order during alveolosphere growth, we compare the three categories of nearest neighbor order z<6,z=6,z>6 across alveolospheres with increasing size to simulations; in simulation, we set particle number N as total cell number and $x_{\text{min}}$ as the average nucleus-to-cell size ratio $d_{n}/L_{\text{av}}$ which are experimentally measured in each alveolosphere. The experimental results are shown as circles and the corresponding simulation results are shown as dashed lines in Fig. 4(b); we find that our simulations successfully replicate the measured nearest neighbor order fractions during alveolosphere growth. This again confirms that the total cell number and nucleus-to-cell size ratio regulate cell packing order during human lung alveolospheres growth.

## V. STRUCTURAL TRANSITION OF ALVEOLOSPHERES FROM GASLIKE TO LIQUIDLIKE STATE

**FIG. 9.**
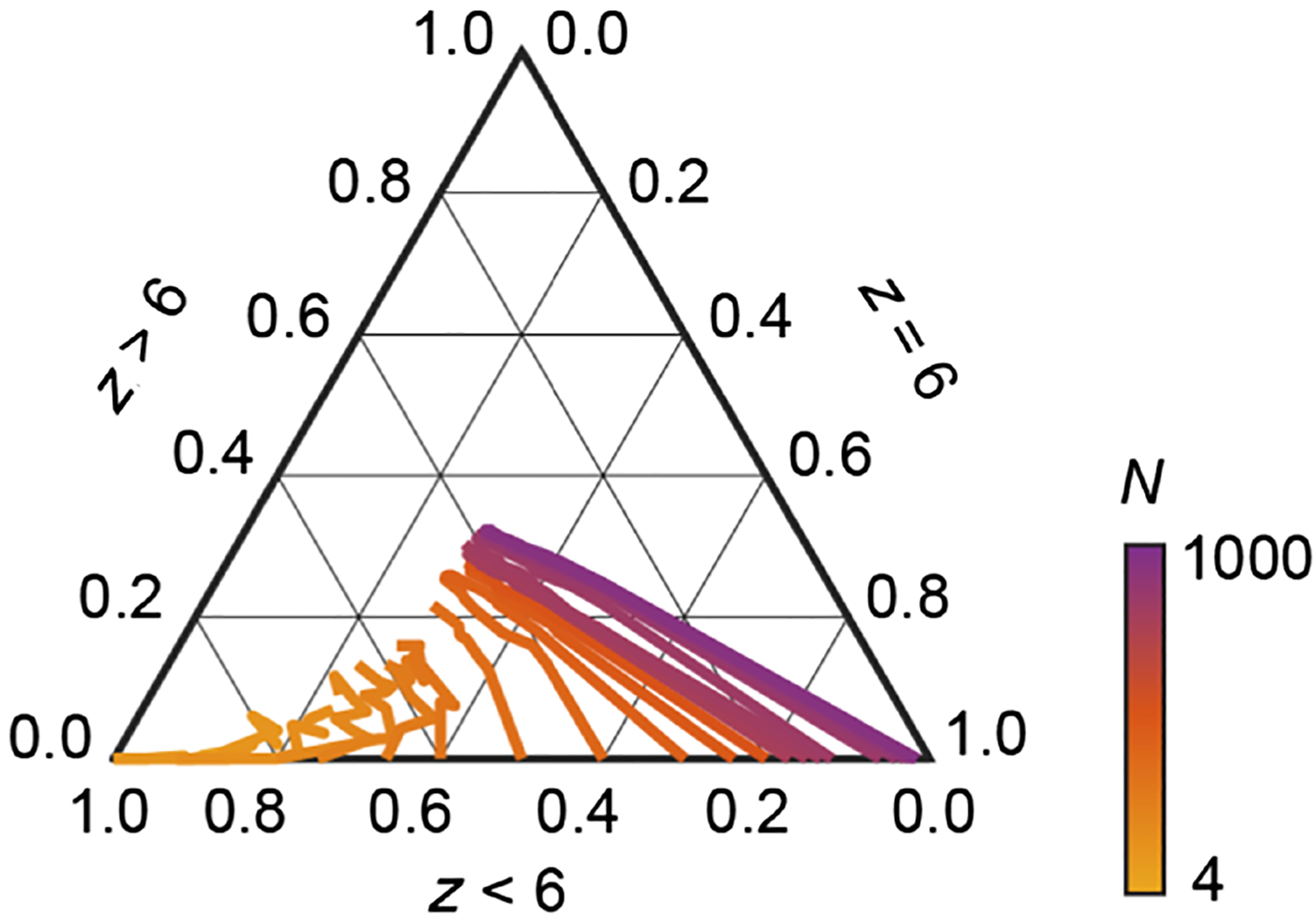
Ternary phase diagram of as a function of N.

In the particle-on-sphere simulation, we examine the radial distribution function g(s) for systems with different minimum dimensionless cell-cell distance $x_{\text{min}}$ [Appendix H; see Fig. 2(e)]. Interestingly, when the particles are randomly packed ($x_{\text{min}}=0$ and thus there is no limitation to the distance between particles), g(s) has similar features as an ideal gas without any apparent peaks, indicating there is no interaction between particles; when $x_{\text{min}}$ becomes large ($x_{\text{min}}=0.7),g(s)$ develops the structure of liquid with a small number of peaks and valleys at the small distances, indicating short-range interaction between particles [33–38]. Thus, the radial distribution function captures a structural gas-to-liquid transition as the dimensionless minimum distance $x_{\text{min}}$ increases [Fig. 2(e)]. To quantitatively study this structural transition, we define a critical transition $x_{c}$ as the $x_{\text{min}}$ when a first peak in g(s) arises (Appendix J). Therefore, for different system size N from small (N=20) to large (N=1000), we can obtain a series of $x_{c}$ forming a gas-liquid phase boundary line (Fig. 11). To correlate this emergent structural transition with the packing order, we plot a ternary phase diagram showing the coexistence of three nearest neighbor orders: z<6,z=6, and z>6 [Figs. 4(d) and 9] and the corresponding $x_{\text{min}}$ and system size N values. Each point in the ternary diagram corresponds to the nearest neighbor order composition at a certain system size N and $x_{\text{min}}$. Figure 9 visualizes the contour lines of constant N with varying $x_{\text{min}}$; from the bottom left to middle of the triangle, N increases. This agrees with the observation that when N is small, there are more cells with z<6, and when N is large, there are more cells with z=6 and z>6 [Fig. 1(d)]. Figure 4(d) visualizes the contour lines of constant $x_{\text{min}}$ with varying N and filled with colors in between (colors are labels according to the values of $x_{\text{min}}$ as shown in the caption); from left to bottom right $x_{\text{min}}$ increases, showing that when $x_{\text{min}}$ increases there are more hexagons. The gas-liquid phase boundary that we obtained from g(s) with Fig. 11 can thus be plotted on the ternary phase diagram [dashed line in Fig. 4(d)].

**FIG. 10.**
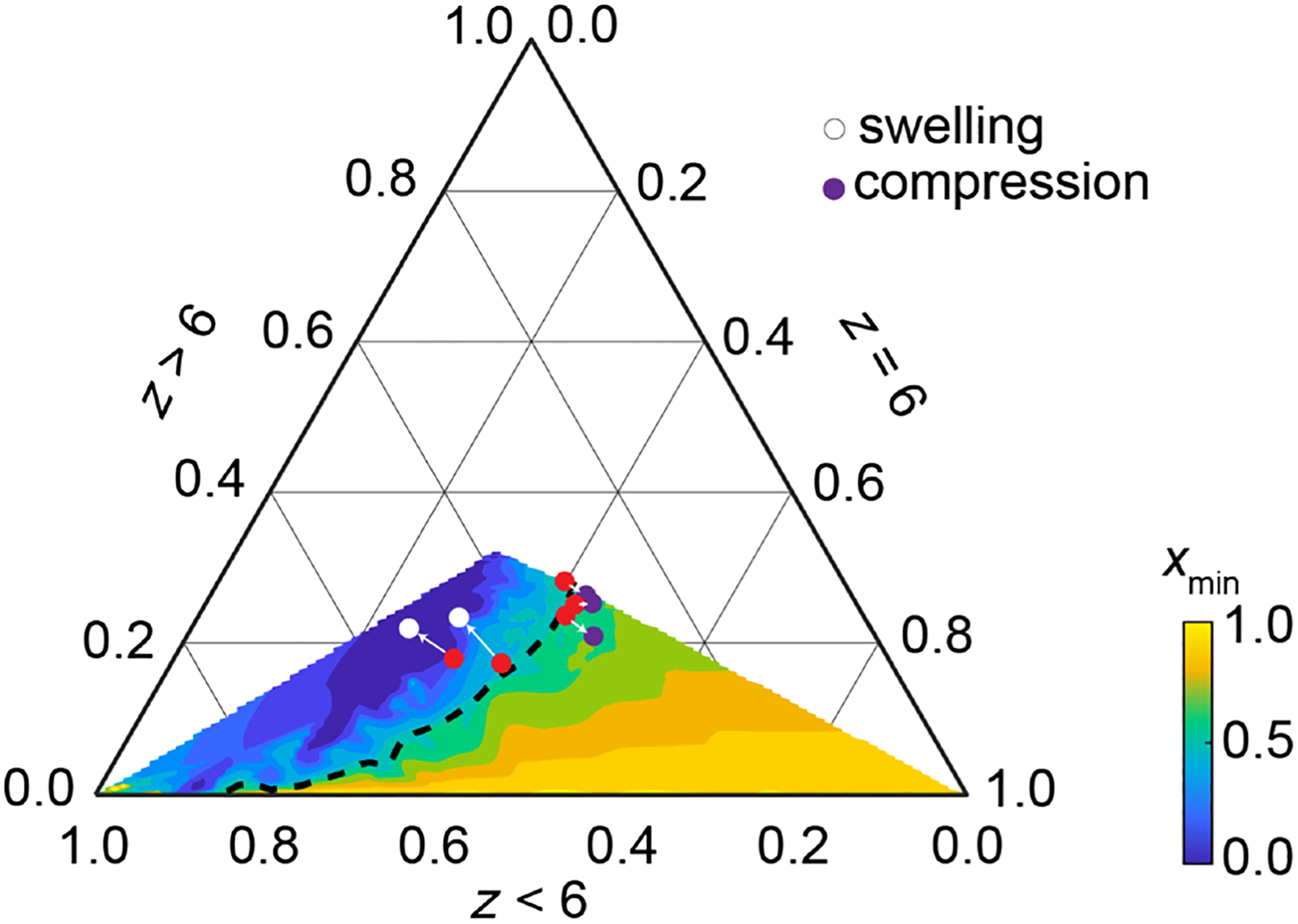
The ternary phase diagram showing the phase change before and after osmotic swelling and compression. Red dots, control; white dots, swelling; purple dots, compression.

**FIG. 11.**
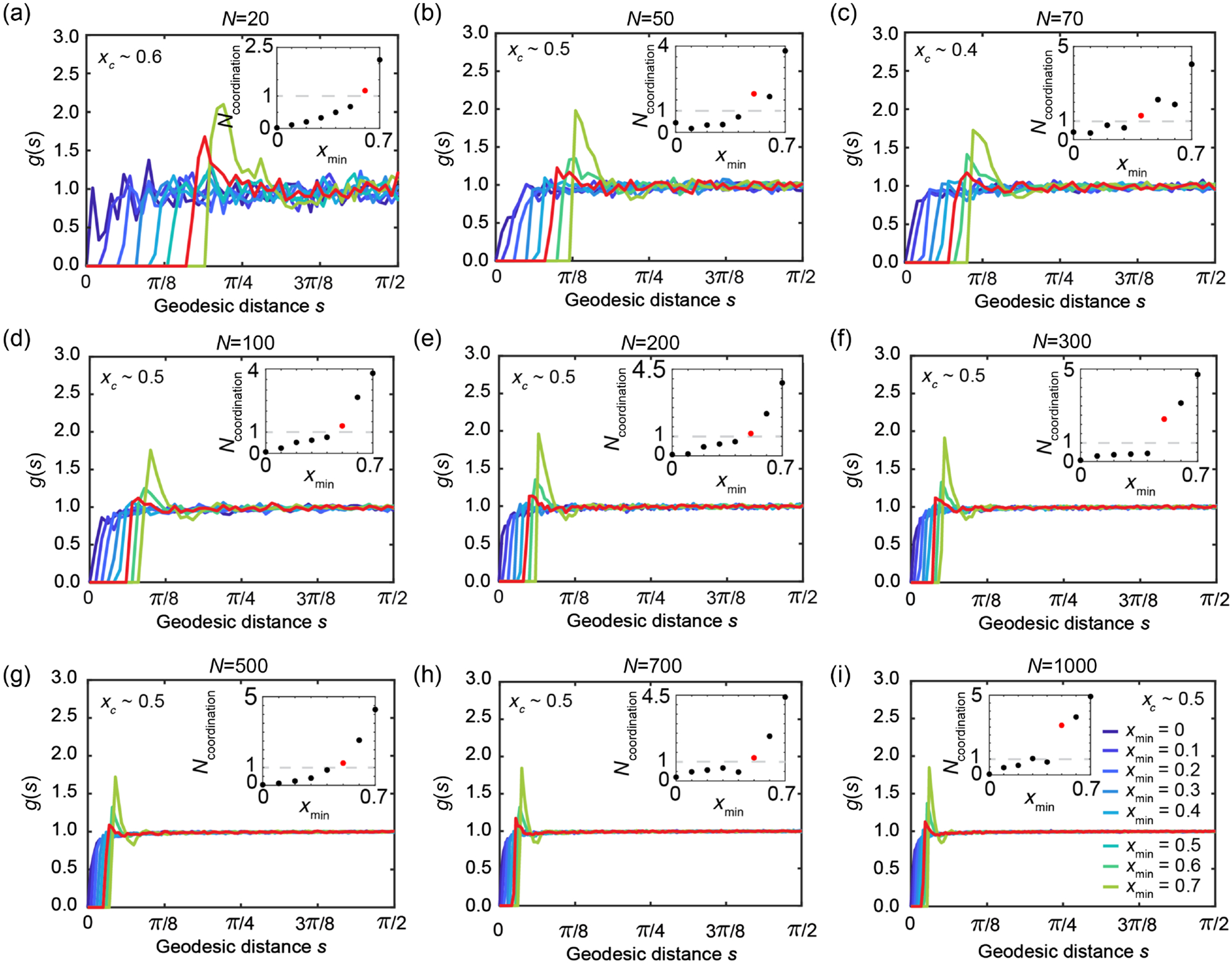
Radial distribution function (RDF) and coordination number for different minimum particle-particle distance ratios. Total particle number: (a)–(i) N=20,50,70,100,200,300,500,700,1000. Inset: coordination number as a function of $x_{\text{min}}$. From the radial distribution function, we can see a transition from gas to liquid as $x_{\text{min}}$ increases. We thus estimate $x_{c}$ for the series of N when the average coordination number starts to become larger than 1, as shown in red RDF and red dots in the insets.

As $x_{\text{min}}$ in the simulation has been confirmed to align well with $d_{n}/L_{\text{av}}$ experimentally measured in alveolospheres, we thus overlay the nearest neighbor order compositions (z<6,z=6, and z>6) of the growing alveolospheres on the ternary phase diagram obtained from simulation [Fig. 4(d)]. Each red dot in Fig. 4(d) represents a nearest neighbor composition of a certain alveolosphere with a system size N and nucleus-to-cell size ratio. As alveolospheres grow bigger, we have shown that there is an increasing ratio of hexagons [Fig. 1(e)]; interestingly, we also notice that the nearest neighbor order compositions of growing alveolospheres pass across the defined gas-liquid phase boundary [Fig. 4(d)]. The idea that a gas-to-liquid structural transition is occurring as the alveolosphere develops is consistent with changes in the radial distribution function: As alveolospheres become bigger, they exhibit significant short-range interactions among neighboring cells [Fig. 4(c)]. This finding suggests that the growing alveolospheres undergoes a gas-to-liquid structural transition. The increase of system size N causes an obvious increase of hexagon fractions in the range of smaller-sized systems (approximately N<200); in larger systems (approximately N>200), the increased topological order is mainly driven by the naturally increasing $d_{n}/L_{\text{av}}$ as this alveolosphere system grows bigger and cells become denser. To further test this, we manipulate the nucleus-to-cell size ratio by applying hyperosmotic pressure or hypo-osmotic pressure, respectively; we indeed find that the multicellular state can be pushed from the gas-liquid boundary either to a more liquidlike state or to a more gaslike state (Fig. 10). Moreover, a material structural transition should also impact dynamic behavior of the constituent particles. To test this behavior, we examine the cell dynamics of the large and the small alveolospheres on two sides of the phase boundary by plotting the cell trajectories over 150 min and comparing the span of these trajectories when putting their initial positions to the center of the coordinates; a larger span suggests a faster cell migration. We find that cells are less motile in the fluid phase compared to gas phase [Figs. 4(e) and 4(f)]; this is also in agreement with our previous observations of less dynamic multicellular flow on larger alveolospheres [19].

## VI. DISCUSSION

In conclusion, using human iPSC-derived lung alveolospheres as a model system, we observe an emergence of cell packing order during an *in vitro* lung spheroids developmental process. Surprisingly, the active cells packing on alveolospheres resembles hard-disks packing on spheres, where nuclei act like effective hard disks surrounded by deformable cytoplasm; both topology and nucleus-to-cell size ratio determine the cell packing order. Remarkably, we find that the area fraction of cell nuclei increases during the growth of lung alveolospheres, which results in the progressive emergence of packing order, more hexagonal cells, and a corresponding gas-to-liquid structural transition.

Not only on 2D spherical epithelia, this increase in nucleus-to-cell ratio has also been observed in 3D tissue during the development of preimplantation mouse embryos from single cell to blastocyst [39] and the starfish embryos [40], suggesting that this might be a general phenomenon to trigger better cell packing during morphogenesis. Recently, nuclear jamming has been found to regulate tissue mechanics and architecture in *in vivo* 3D *Drosophila* brain and retina [17], where a higher nucleus-to-cell area ratio and a better cell packing were observed. Furthermore, increased shape index indicating a worse cell packing has recently been shown to correlate to increased tissue fluidity in cancerous cell spheroids as compared to the benign spheroids in 3D [6], where the relative nuclear-to-cell size ratio seems to also increase based on the reported images. The nucleus, considered as the control center in eukaryotic cells, contains the genetic information of life. Being able to regulate its relative size to the cell size is important to control the intranuclear environment (e.g., the degree of crowding) and thus a myriad of biological functions [39–46]. For example, since the stiff cell nucleus accounts for a larger portion of the cell during morphogenesis, it can potentially contribute to an increase in epithelial rigidity that facilitates stabilization of tissue geometry and thus maturation. Indeed, as these iPSC-derived alveolospheres grow bigger, we observe that cell migration slows down, resulting in a more stabilized cell neighborhood; this potentially can promote a stronger and more stable cell-cell communication and thus influence tissue biological functions. Finally, the fundamental rules of how soft living cells tile curved tissue surfaces may have a broad implication to designing soft robotics, wearable sensors, as well as metamaterials.

## Data Availability

Our simulation code is available at GitHub [47]. The data that support the findings of this article are openly available [48].

## APPENDIX A: ALVEOLOSPHERES 3D CULTURE

Human induced pluripotent stem cell–derived alveolar epithelial type II cells were generated via directed differentiation as previously described [49,50]. Two iPSC lines were employed as indicated in the text: clone SPC2-ST-B2 and clone BU3 NGST. After differentiation into iAT2s, cells were maintained in 3D Matrigel (Corning no. 354230) as “alveolospheres” at 400 cells/μl Matrigel, and passaged every 10–14 days, as published [28,49,50]. A lentiviral vector with constitutively and ubiquitously active long EF1a promoter (EF1aL) driving expression of nuclear localized GFP (nlsGFP) was engineered by cloning a nuclear localizing sequence (NLS) in front of GFP. The resulting construct (pHAGE-EF1aL-nlsGFP-W) with full plasmid map and sequence are available from Addgene, plasmid no. 126688. Lentiviral infection of iAT2s was performed in suspension culture at a multiplicity of infection of 20, as previously detailed [50], and NLS GFP expressing cells were purified at a subsequent culture passage through fluorescence activated cell sorting and replated for serial sphere passaging in 3D culture. For imaging, NLS GFP–expressing iAT2s were seeded in 3D Matrigel droplets in 35-mm glass-bottom petri dishes at 100–200 cells/μL. These cells grow, proliferate, and self-organize into alveolospheres in 3D conditions.

## APPENDIX B: TOPOLOGICAL CONSTRAINT ON SPHERES

The nearest neighbor order for each cell is defined by its vertex number $z_{i}$, and the average cell vertex number on each alveolosphere is $\left\langle z_{i}\right\rangle$. In the theory in colloidal crystallization [25,27], cells that do not have six vertexes are defined as topological defects, and the corresponding defect charge for each defect cell is defined as $q_{i}=6-z_{i}$. For a given system, we calculate the total topological charge $Q=\sum_{i=1}^{N}\left(6-z_{i}\right)$, where N is the total cell number on an alveolosphere [1,25]. For a sphere, a total +12 charges are required by a classical theorem of Euler equating the total disclination charge to 6χ. For any polyhedron or surface that is homeomorphic to a sphere, Euler characteristic

(B1)
χ=V-E+F=2,

where V is the number of vertices, E is the number of edges, and F is the number of faces [1,25,26]. In Voronoi tessellation, if the facets meet at three valanced vertices, then we have

(B2)
$$V=\frac{1}{3}\sum_{k}kN_{k},$$

where k is the number of edges for a Voronoi cell, $N_{k}$ is the number of Voronoi cells with k edges, divided by 3 because each vertex is shared by three cells. The total number of edges

(B3)
$$E=\frac{1}{2}\sum_{k}kN_{k},$$

where the factor $\frac{1}{2}$ comes from the fact that each edge is shared by two adjacent cells. The total number of facets F equals to the total number of cells N. Therefore, the Euler’s polyhedron formula becomes

(B4)
$$\frac{1}{3}\sum_{k}kN_{k}-\frac{1}{2}\sum_{k}kN_{k}+N=2$$

and simplifies the equation

(B5)
$$\sum_{k}kN_{k}=6N-12,$$

where the left-hand side represents the total number of neighbors across all the cells. We divide both sides of the equation by cell number N to find the average vertex number of each cell $z_{\text{av}}$, as

(B6)
$$z_{\text{av}}=6-\frac{12}{N},$$

where the average vertex number for cells is a function of total cell number N due to the topological constraint and the requirement that the facets meet at three valanced vertices. Other topological constraint can be applied to different tissues with different geometry and structures.

## APPENDIX C: PLATEAU OF NEAREST NEIGHBOR ORDER FRACTIONS AT LARGE N

All the three categories of nearest neighbor order fractions reach plateaus when N is large, indicating that the population size N strongly affects nearest neighbor order fractions only on small alveolospheres, while its effect is diluted when N is large. The dominance of N is evident with more variations in nearest neighbor order fractions at small system size (Fig. 5). As we compare these results with a random particle-on-sphere simulation on a sphere, we find that although they both have plateaus at large N, the three compositions of cell nearest neighbor order (z<6,z=6,z>6) are significantly different (Fig. 5, inset). This difference in nearest neighbor order suggests that cell packing on spherical surfaces is not entirely random, and the topological constraint is not the only factor that decides cell arrangement in spherical epithelia.

## APPENDIX D: EFFECTIVE PACKING FRACTION

Assume $x_{\text{min}}$ corresponds to a hard sphere diameter ${\left(D_{p}\right)}_{\text{min}}=x_{\text{min}}L_{\text{av}}$ where the hard spheres cannot overlap, i.e., the distances between hard spheres must be larger than $x_{\text{min}}L_{\text{av}}$. According to the definition,

(D1)
$$\phi =\frac{\text{hard}\;\text{disk}\;\text{area}}{\text{total}\;\text{surface}\;\text{area}},$$

we have

(D2)
$$\phi =\frac{N\pi {\left(\frac{{\left(D_{p}\right)}_{\text{min}}}{2}\right)}^{2}}{4\pi R^{2}}=\frac{N{\left[x_{\text{min}}\sqrt{\frac{4\pi R^{2}}{N}\left(\frac{2}{\sqrt{\left(3\right)}}\right)}\right]}^{2}}{16R^{2}}=x_{\text{min}}^{2}\frac{\pi }{2\sqrt{3}},$$

where ϕ is only a function of $x_{\text{min}}$. Therefore, when in range [0, 0.7], packing fraction is in a range of [0, 0.444]. This range is smaller compared to the maximum packing fraction for random packing of hard disks with the same size, which ϕ=0.85.

## APPENDIX E: PARTICLE-ON-SPHERE SIMULATION

In the simulation of randomly placing N particles on a unit sphere, the spherical coordinates (θ,ϕ,R) are randomly generated using the uniformly distributed “rand” function in matlab. Specifically, θ=π× rand and ϕ=2π× rand. The spherical coordinates are then converted to Cartesian coordinates for Voronoi tessellation. The particle-on-sphere simulation is performed using the random sequential addition method on unit spheres. Basically, particles are randomly generated with spherical coordinates on a unit sphere one by one, and then the spherical coordinates are converted into Cartesian coordinates. The newly generated particle will be kept on the sphere if the distances between it and its nearest neighbors are within the set value: ${\left(D_{p}\right)}_{\text{min}}=x_{\text{min}}L_{\text{av}}$. Otherwise, it will be removed, and a new particle will be generated again at a random position on sphere. This process will loop until the total number of particles reaches to the set total system size N. When the loop ends, there will be N particles sitting on the sphere and the distance between any nearest neighbor particles is larger than what we set. To make comparison between different system sizes, we nondimensionalize $D_{p}$ by average cell size $L_{\text{av}},x_{\text{min}}={\left(D_{p}\right)}_{\text{min}}/L_{\text{av}}$. And we vary $x_{\text{min}}$ for different cases in simulation.

## APPENDIX F: CHANGE OF $L_{\text{av}}$ AND $d_{n}$ DURING OSMOTIC PERTURBATIONS

Osmotic perturbations are performed using established methods as described in previous literature [5,29,30]. Osmotic compression is applied by adding 1.5 Vol./Vol. % PEG300 to isotonic culture medium in order to squeeze the alveolospheres. Osmotic swelling is applied by adding 20 Vol./Vol. % deionized water to isotonic culture medium in order to swell the alveolospheres. As shown in Figs. 8(a) and S5(b), both the smallest nuclear dimension $d_{n}$ and average cell size $L_{\text{av}}$ decrease after osmotic compression. Similarly, both $d_{n}$ and $L_{\text{av}}$ get larger after osmotic swelling [Figs. 8(c) and S5(d)]. However, in both cases the average cell size change is larger than the nuclear size change. Therefore, we observe an increase of the ratio $d_{n}/L_{\text{av}}$ during osmotic compression, and a decrease of $d_{n}/L_{\text{av}}$ during osmotic swelling.

## APPENDIX G: BOND ORIENTATIONAL ORDER $\left|\psi_{6}\right|$

The bond orientational order at each cell with position $r_{i}$ is calculated following the equation $\psi_{6}\left(r_{i}\right)=\left(1/N_{i}\right)\sum_{j=1}^{N_{i}}e^{6i\theta_{ij}}$, where $N_{i}$ is the coordination number of the ith cell, and $\theta_{ij}$ is the angle between the line connecting cell i to its jth neighbor and an arbitrary reference axis. On a flat surface, the largest $\left\langle |\psi_{6}|\right\rangle$ can reach 1; however, on a sphere, $\left\langle |\psi_{6}|\right\rangle$ reaches a plateau of ~0.87. Bond orientational order matlab code was adapted from Ref. [12]. When estimating $\left|\psi_{6}\right|$ on a curved surface, the calculation for each particle is performed within a single tangent plane.

## APPENDIX H: RADIAL DISTRIBUTION FUNCTION g(s)

The radial distribution function is calculated along the geodesic distance. The density variation is first calculated for each particle before taking an ensemble average,

(H1)
$$g\left(s\right)=\frac{1}{N}\sum_{i=1}^{N}g_{i}\left(s\right),$$

where N is the total cell number and $g_{i}(s)$ is the radial distribution function calculated for each cell using the following formula,

(H2)
$$g_{i}\left(s\right)=\frac{\rho \left(s\right)}{\rho_{\text{bulk}}}\approx \frac{\frac{dn\left(s\right)}{2\pi r\;\text{sin}\;\theta d\left(s\right)}}{\frac{N}{4\pi r^{2}}},$$

where dn(s) is the cell number found within a short geodesic distance range d(s) at s.

## APPENDIX I: TERNARY PHASE DIAGRAM

Ternary phase diagrams of the packing fractions z<6,z=6, and z>6 are plotted as functions of both $x_{\text{min}}$ and total number N. The background color are generated by particle-on-sphere simulation. Each point in the ternary diagram corresponds to the nearest neighbor order composition at a certain system size N and $x_{\text{min}}$. Figure 9 visualizes the contour lines of constant N with varying $x_{\text{min}}$; from the bottom left to middle of the triangle, N increases. This agrees with the observation that when N is small, there are more cells with z<6, and when N is large, there are more cells with z=6 and z>6. Figure 4 (d) visualizes the contour lines of constant $x_{\text{min}}$ with varying N and filled with colors in between (colors are labeled according to the values of $x_{\text{min}}$ as shown in the caption); from left to bottom right $x_{\text{min}}$ increases, showing that when $x_{\text{min}}$ increases there are more hexagons. Ternary phase diagram code is adapted from the matlab code [54].

## APPENDIX J: DETERMINING THE GAS-LIQUID PHASE BOUNDARY

The gas-to-liquid phase boundary is determined using particles-on-sphere simulation. For a series of different system sizes N, we plot the radial distribution functions g(s) with different $x_{\text{min}}$, as shown in Fig. 11. When $x_{\text{min}}$ is small, radial distribution function shows no peak, suggesting there is no interaction between particles like an idea gas. When $x_{\text{min}}$ is large, the radial distribution function shows short-range peak and valley, which is similar to what is observed both experimentally and in simulations for liquids [33–38]. Given these two end points, there must be a transition from ideal gas to liquid in between them. We thus define a critical transition $x_{c}$ here as the $x_{\text{min}}$ when a significant first peak starts to appear in the radial distribution function as we gradually increase $x_{\text{min}}$. Although a single peak in radial distribution function is observed in hard sphere “gas” simulations [35], here we use the appearance of first peak as a signature that the system is no longer ideal gas but starts to show significant particle-particle interaction. To have a quantitative identification, we define the $x_{c}$ as the point at which the integration of g(s) from 0 to the first valley equals 1 [51–53],

(J1)
$$N\left(s^{*}\right)={\left.\int_{0}^{s^{*}}g(s)\right|}_{x_{c}}2\pi \;\text{sin}(s)\left(\frac{N}{4\pi R^{2}}\sqrt{\frac{3}{2}}\right)ds=1,$$

where $s^{*}$ is the geodesic distance for the first valley. $N\left(s^{*}\right)=1$ suggests there is one particle in the nearest neighbor, where $s^{*}$ is the geodesic distance when the first valley appears [51–53]. Then for each system size N we can find a critical $x_{c}$. The series of N and $x_{c}$ correspond to different nearest neighbor compositions on the ternary phase diagram, and we mark the self-defined phase boundary as a black dashed line [Fig. 4(d), right].
